# ADAM10: Possible functions in enamel development

**DOI:** 10.3389/fphys.2022.1032383

**Published:** 2022-11-25

**Authors:** Shifa Shahid, Atsushi Ikeda, Michelle C. Layana, John D. Bartlett

**Affiliations:** Division of Biosciences, College of Dentistry, The Ohio State University, Columbus, OH, United States

**Keywords:** ameloblasts, cell migration, enamel defects, cell surface proteins, sheddase, COL17A1, RELT

## Abstract

ADAM10 is A Disintegrin And Metalloproteinase (ADAM) family member that is membrane bound with its catalytic domain present on the cell surface. It is a sheddase that cleaves anchored cell surface proteins to shed them from the cell surface. ADAM10 can cleave at least a hundred different proteins and is expressed in most tissues of the body. ADAM10 is best characterized for its role in Notch signaling. Interestingly, ADAM10 is transported to specific sites on the cell surface by six different tetraspanins. Although the mechanism is not clear, tetraspanins can regulate ADAM10 substrate specificity, which likely contributes to the diversity of ADAM10 substrates. In developing mouse teeth, ADAM10 is expressed in the stem cell niche and subsequently in pre-ameloblasts and then secretory stage ameloblasts. However, once ameloblasts begin transitioning into the maturation stage, ADAM10 expression abruptly ceases. This is exactly when ameloblasts stop their movement that extends enamel crystallites and when the enamel layer reaches its full thickness. ADAM10 may play an important role in enamel development. ADAM10 can cleave cadherins and other cell-cell junctions at specific sites where the tetraspanins have transported it and this may promote cell movement. ADAM10 can also cleave the transmembrane proteins COL17A1 and RELT. When either *COL17A1* or *RELT* are mutated, malformed enamel may occur in humans and mice. So, ADAM10 may also regulate these proteins that are necessary for proper enamel development. This mini review will highlight ADAM10 function, how that function is regulated by tetraspanins, and how ADAM10 may promote enamel formation.

## Overview of enamel development

**FIGURE 1:**
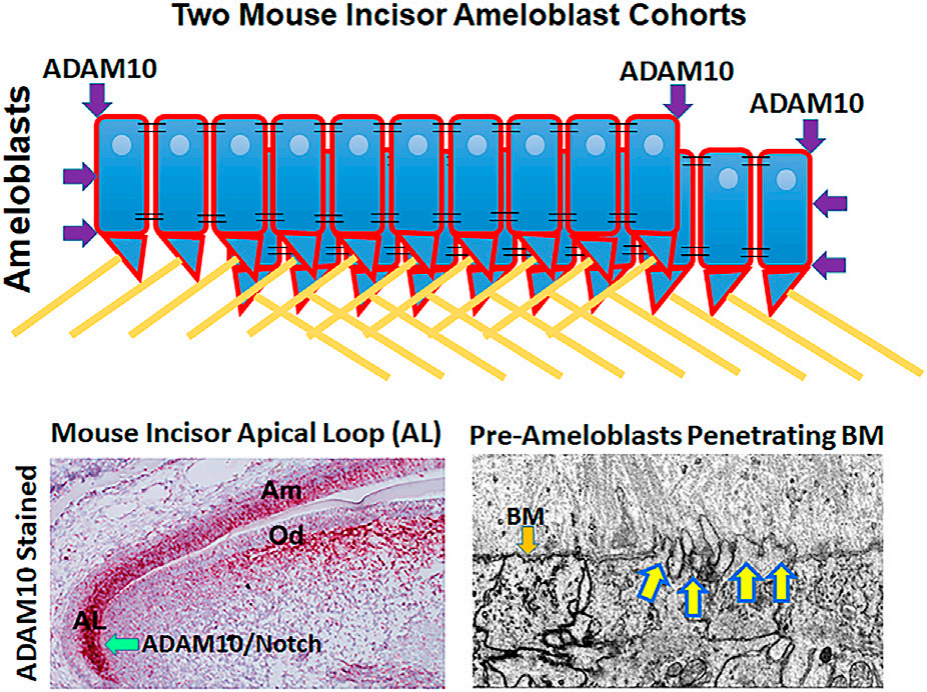
Possible mechanisms by which ADAM10 may facilitate enamel development. (Top) Schematic showing how ADAM10 may cleave the ends of ameloblast cohorts so that they can slide by one another during the secretory stage of enamel development. The cell-cell connections would also be removed between the sliding cells (not shown). Black parallel lines, tight junctions; right triangles, Tomes’ processes; yellow lines, enamel rods (interrods not shown). (Bottom Left) Mouse incisor section stained red by *in situ* hybridization for ADAM10 (Ikeda et al., 2019). *Adam10* expression begins in the apical loop and continues until the ameloblasts reach the transition stage of enamel development. Adam10 may facilitate Notch signaling to maintain the stem cell niche within the apical loop (Felszeghy et al., 2010). (Bottom Right) Focused Ion Beam Scanning Electron Microscopy (FIB-SEM) demonstrating that pre-ameloblasts extent finger-like projections to penetrate the basement membrane (BM) that separates the pre-ameloblastss from the predentin organic matrix (Bartlett et al., 2021). Since ADAM10 is expressed during this time, it is possible that it assists in penetrating the basement membrane.

In the continuously erupting mouse incisor, enamel development starts with the stem cell niche in the apical loop that is furthest away from the incisor eruption point (Figure 1). Stem cells migrate out of the loop, develop into pre-ameloblasts and begin their journey moving distally to where the incisor erupts. The pre-ameloblasts form a single layer aligned along a basement membrane and as they move distally in the direction of eruption, they pass through defined developmental stages. First, the pre-dentin matrix begins to form and as development progresses, the pre-ameloblasts extend finger-like protrusions that penetrate the basement membrane and move in between the pre-dentin collagen fibrils.

At approximately this point, the pre-ameloblasts become secretory stage ameloblasts because they initiate the secretion of amelogenin. Eventually the entire basement membrane is removed and after this, the pre-dentin begins to mineralize starting near the ameloblast layer. Once the dentin near the ameloblasts is mineralized, ameloblasts start the growth of enamel ribbons that appear to initiate on the mineralized collagen fibers within the dentin. These ribbons extend and are intimately associated with the ameloblast cell membrane. The initial enamel ribbons are the beginning of the developing interrod enamel. The ameloblasts move back to allow the ribbons to grow in length. As this occurs, space is created to allow the ameloblasts to form Tomes’ processes that then initiate rod enamel ribbon formation. The rod and interrod enamel ribbons are within a few degrees of being at right angles to one another (Bartlett et al., 2021).

Secretory stage ameloblasts will eventually define the full thickness of the developing enamel layer. It is during this stage that amorphous ribbons convert into crystallites and when large amounts of enamel proteins are secreted into the enamel matrix. These proteins include amelogenin, ameloblastin, enamelin, and matrix metalloproteinase-20 (MMP20, enamelysin). It is during the secretory stage that cohorts of ameloblasts slide by one another to begin forming the decussating (crisscrossing) enamel rod pattern characteristic of mouse incisor enamel. How these cohorts of ameloblast stay attached to each other while detaching from adjacent cohorts remains a mystery.

Another mystery is in understanding why deletion or mutations in Plectin (*PLEC*), Laminin 332 (*LAMA3*, *LAMB3*, and *LAMC2*), COL17A1, and α6ß4 (*ITGA6* and *ITGB4*) cause enamel malformations. These proteins combine to form an attachment complex in skin. It was postulated (Simmer et al., 2021) that these proteins also serve to attach enamel ribbons at the ameloblast membrane by forming a complex that may bind enamel matrix proteins necessary for ribbon growth, such as binding to enamelin and/or ameloblastin. This could allow the ameloblasts to lengthen the ribbons as they move back to facilitate crystallite growth. In support of this postulate, full-length, uncleaved enamelin is only found abutting the ameloblast membrane and if enamelin is deleted from the mouse genome, no enamel, and virtually no mineral forms, near the dentin (Hu et al., 2014). And, previous studies have demonstrated that laminin 332 is necessary for ameloblast attachment to the underlying secretory stage enamel (Sahlberg et al., 1998; Ryan et al., 1999).

As the secretory stage nears its end, the enamel is protein-rich and has a soft cheese-like consistency. However, this dramatically changes as the secretory stage ameloblasts transition into maturation stage ameloblasts. The tall columnar secretory stage ameloblasts become reduced in height and start secreting kallikrein-related peptidase 4 (KLK4), which cleaves the enamel matrix proteins to facilitate their export out of the enamel matrix. It is during the maturation stage of enamel development that the enamel crystallites grow in width and thickness to form a virtually protein free enamel layer that is the hardest most mineralized substance in the body. After this stage the ameloblasts become reduced in size and cover the hardened enamel as tooth eruption occurs (Bartlett, 2013).

## Introduction to ADAMs

ADAMs are a family of metalloproteinases characterized by having A Disintegrin And Metalloproteinase domain. They also contain a cysteine rich domain followed by a transmembrane region and a cytoplasmic tail. Therefore, the ADAMs are transmembrane proteins with their catalytic domains located outside the cell near the cell surface where they cleave the extracellular domains of membrane bound proteins. This process is termed ectodomain shedding (Reiss and Saftig, 2009).

**FIGURE 2:**
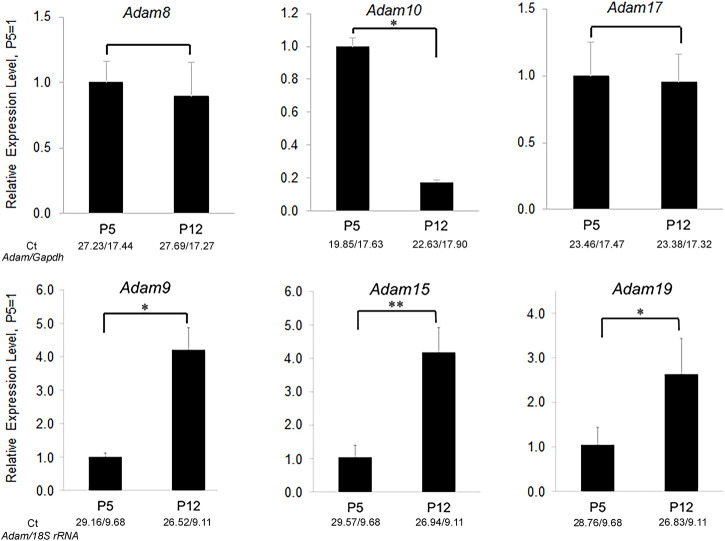
Relative expression levels of ADAM family members in murine first molar enamel organs from P5 (secretory stage) and P12 (maturation stage) mice. qPCR demonstrated that *Adam8, 9, 10, 15, 17*, and *19* were expressed in mouse enamel organs (Ikeda et al., 2019). However, only *Adam10* was expressed predominantly in the secretory stage when ameloblast movement occurs. Three biological replicates were analyzed for each of three experiments (*, *p* < 0.05; **, *p* < 0.01; Ct, cycle threshold).

ADAMs have an N-terminal pro-domain that covers an otherwise active catalytic domain. Its removal by proteolysis results in ADAM catalytic activation. ADAMs cleave type I and Type II transmembrane and glycosylphosphatidylinositol (GPI)-anchored proteins near the plasma membrane (Wetzel et al., 2017). ADAMs have been implicated in numerus physiological roles including, cell signaling, development, wound healing, inflammation, and migration. Twenty two ADAMs have been identified in humans. However only twelve are capable of becoming catalytically active and the other ten have non-functional catalytic domains and are therefore not as well studied (Harrison et al., 2021). Previously we performed an active ADAM screen of the mouse enamel organ and discovered that ADAM10 was the only ADAM expressed predominantly in early, but not late enamel development (Figure 2).

Here we will review the functional characteristics of ADAM10, show what is known about ADAM10 in enamel development, and present evidence about ADAM10’s possible mechanistic function during enamel formation.

## ADAM10 overview

ADAM10 starts as an inactive zymogen and is activated in the trans-Golgi network by two pro-protein convertases that cleave the ADAM pro-domain (Vincent, 2016). Additionally, ADAM10 is glycosylated in four places in its extracellular domain and these glycosylations ensure proper trafficking and stability (Vincent, 2016). A select group of six different tetraspanins can each traffic ADAM10 from the endoplasmic reticulum, through the Golgi and to the cell surface. Depending on the tetraspanin involved, ADAM10 will localize to different subcellular locations. Therefore, tetraspanins can direct ADAM10 to the substrates it may cleave and ADAM10 is known to have over 100 substrates (Harrison et al., 2021). However, its proteolytic activity can be inhibited by both tissue inhibitor of metalloproteinase-1 and -3 (Timp1 and Timp3) (Giebeler and Zigrino, 2016). Since *Adam10*-ablated mice die at embryonic day 9.5, ADAM10 is essential for embryonic development (Weber et al., 2011).

Human ADAM10 haploinsuficiency causes the rare hyperpigmentation skin disease named reticulate acropigmentation of Kitamura (Kono and Akiyama, 2019). To date no other defects including enamel defects are reported in these patients, likely indicating that for all other organs ADAM10 haploinsuficiency is adequate for proper development.

## ADAM10 and the Notch pathway

The best characterized function of ADAM10 is in Notch signaling. ADAM10 cleaves/sheds Notch proteins, which subsequently allows the remaining truncated membrane tethered Notch fragment to undergo intramembrane proteolysis by a ɣ-secretase. This allows translocation of the Notch intracellular domain into the nucleus where it can function as a transcriptional regulator (Matthews et al., 2017). ADAM10 deficient mice have similar defects as Notch knockout mice, such as CNS, somite and cardiovascular defects, which suggests a major role for ADAM10 in Notch signaling (Hartmann et al., 2002). Notch pathway loss-of-function phenotypes are also observed when ADAM10 is deleted from epithelial tissues (Weber et al., 2011).

Ameloblasts are the cells responsible for enamel formation and Notch proteins may maintain their stem cell niche in the apical loop of the continuously erupting mouse incisor (Felszeghy et al., 2010). However, Notch 1, 2, and 3 are not expressed by both pre-ameloblasts and ameloblasts (Mitsiadis et al., 1995), which are the cells that initiate and facilitate enamel formation.

## ADAM10 and cell migration

Cancer cell migration and physiological migration, such as T-cell migration through endothelial cell layers are well documented to be facilitated by ADAM10 (Reiss and Saftig, 2009). The vast majority of ADAM10 literature points to ADAM10 shedding substrates from the cell surface on the same cells where both ADAM10 and the substrates reside. These substrates include cell-cell binding proteins such as E-, N- and VE-cadherins (Dempsey, 2017) and tight junction proteins such as F11R and JAM3 (Wetzel et al., 2017). However, evidence exists that ADAM10, either directly or indirectly, cleaves substrates that are not specifically present on a cell surface. For example, Type IV collagen is a major component of basement membranes and ADAM10 was demonstrated to cleave Type IV collagen *in vitro* (Millichip et al., 1998). ADAM10 also facilitates the migration of bone marrow derived mast cells (BMMC) through collagen IV-coated transwells when compared to *Adam10* ablated BMMCs (Faber et al., 2014). At least two other studies have demonstrated, by use of transwell assays with matrigel coated filters, that active ADAM10 facilitates cell migration/invasion through these coated filters (Ikeda et al., 2019; Sun et al., 2019).

Additionally, ADAM10 overexpression *in vivo* in a mouse emphysema model was responsible for the destruction of lung parenchyma with associated basement membrane proteolysis. Note that Type IV collagen is the most abundant non-fibril forming collagen in lung tissue and is present in both alveolar and capillary basement membranes. ADAM10 was overexpressed by use of an adenovirus gene transfer vector coding for human ADAM10 that was introduced intratracheally into mice. Assessment of their lungs after 2 months, demonstrated enlarged airspaces, as would occur with emphysema. The authors cautioned that ADAM10 could have acted by directly destroying the lung tissue or may have acted indirectly by causing inflammation and/or apoptosis (Saitoh et al., 2009).

## ADAM10 and tetraspanins (Tspans)

Tetraspanins are a superfamily of 33 membrane proteins (Tspan1–Tspan33) in mammals, which transport and regulate partner proteins containing transmembrane domains. In addition to transporting proteins from the ER through the Golgi and to the cell surface, Tspans also bind with partner proteins that influence Tspan intracellular trafficking, clustering, lateral mobility and compartmentalization (Matthews et al., 2018). Once at the cell surface, Tspans form small assemblies of approximately ten members of a single type per cluster (Harrison et al., 2021).

A subset of Tspans are the TspanC8s, which contain eight cysteine residues in their extracellular domains. Six different TspanC8s (Tspan 5, 10, 14, 15, 17, and 33) transport and regulate ADAM10 at the cell surface. Additionally, TspanC8s will localize ADAM10 to specific subcellular domains and astonishingly, can influence ADAM10 substrate specificity. This was first observed when it was discovered that Tspan15/ADAM10 is associated with N-cadherin shedding (Harrison et al., 2021). It also fits with the observation that ADAM10 substrate preferences have little correlation with the protein sequence of their substrates (Matthews et al., 2017). It is unclear precisely how TspanC8s modulate ADAM10 substrate specificity. Mechanisms may include subcellular localization, direct interaction with substrates and/or modulation of ADAM10 conformation (Harrison et al., 2021). Therefore, ADAM10 likely has six different sets of substrate specificities depending on the TspanC8 it associates with and this likely contributes to its diverse substrate repertoire of over 100 substrates. Interestingly, it was previously suggested that since ADAM10 can be delivered by tetraspanins to specific cell surface locations, that this may be a way for ADAM10 to specifically separate cell-cell connections between cohorts of ameloblasts that move in opposite directions. Therefore, the tetraspanins may allow ADAM10 to cleave cell-cell connections between adjacent cohorts but leave the cohorts intact (Ikeda et al., 2019).

## ADAM10 substrates pertinent to enamel formation

Since ADAM10 expression initiates in the apical loop of the mouse incisor (Figure 1) and its expression continues throughout the secretory stage of enamel development, ADAM10 may participate in growth of the enamel crystallites until they lengthen to form the full thickness of the developing enamel. Below are proteins that are shed by ADAM10 and that are expressed by ameloblasts. When mutated, these proteins can cause enamel malformation.

### Type XVII collagen

Collagen XVII (COL17A1) is a homotrimer of three alpha1-XVII chains. It is a type II transmembrane protein that is a member of the superfamily of transmembrane collagens. Originally it was discovered in epithelial cell hemidesmosomes where it plays an important role in hemidesmosome stability and epithelial attachment (McGrath et al., 1995). Although functionally not well characterized, it is also present in other tissues in a hemidesmosome-independent manner, such as in the brain, kidney, placenta (Tuusa et al., 2021) and enamel organ.

*COL17A1* deletion or mutations can cause non-Herlitz junctional epidermolysis bullosa (JEB), which manifests when the epidermis separates from the dermis (Tuusa et al., 2021). Patients with *COL17A1*-mediated JEB typically also have enamel malformations characterized by enamel hypoplasia and pitting of the enamel surface (Asaka et al., 2009). Of interest is that COL17A1 is expressed during the secretory stage of enamel development (Simmer et al., 2021) when ADAM10 is also expressed. Both ADAM9 and ADAM10 were demonstrated to shed COL17A1 from the cell surface of keratinocytes (Franzke et al., 2009). However, in contrast to ADAM10, little to no ADAM9 is expressed in mouse molar enamel organs that are predominantly in the secretory stage of enamel development (Figure 2). Therefore, COL17A1 could be an ADAM10 substrate of interest during the early stages of enamel formation.

A possible mechanism of how ADAM10 may function to promote enamel development could involve its shedding of COL17A1 from ameloblasts. Perhaps, as previously proposed (Simmer et al., 2021), COL17A1 is a component of the apparatus that attaches the forming ribbons to the retreating ameloblasts as the ribbons grow in length. If ribbon growth requires the disengagement and then reattachment of the ribbons to the apical end of ameloblasts, then it may be possible that ADAM10 functions to promote the detachment process by cleaving COL17A1, which could dislodge the ameloblasts from the lengthening ribbons and subsequently allow the ameloblasts to reattach at a site distal to the previous attachment site. However, this postulated mechanism will need to be experimentally validated.

### Type IV collagen

Currently it is unknown how the basement membrane between pre-ameloblasts and the pre-dentin matrix is removed. Ameloblasts have finger-like projections that protrude through the basement membrane as they initiate its removal. So, the possibility exists that a membrane bound proteinase would help the projections to penetrate the basement membrane. ADAM10 is expressed in the mouse incisor apical loop well before the basement membrane is degraded and its expression persists well after the basement membrane is removed. Perhaps, ADAM10 could pair with a different tetraspanin to degrade the basement membrane Type IV collagen and switch to another tetraspanin once the membrane is removed. However, this supposition needs to be demonstrated.

### RELT

RELT is a member of the tumor necrosis factor receptor superfamily (TNFRSF). The TNFRSF members are transmembrane proteins that regulate biological processes such as cell death, inflammation plus cell differentiation and development. RELT is highly expressed in lymphoid tissues, hence it was named Receptor Expressed in Lymphoid Tissues. An interesting aspect of RELT is its designation as an orphan receptor because to date, no ligand has been identified that binds to RELT. However, *RELT* mutations cause autosomal recessive amelogenesis imperfecta where the dental enamel is of normal or near normal volume in unerupted teeth, but is hypomineralized, which causes the enamel to abrade from erupted teeth. This phenotype is typically indicative of a maturation stage defect. However in mice, *Relt* is only expressed on pre-ameloblasts and secretory stage ameloblasts, but not on maturation stage ameloblasts (Kim et al., 2019). Moreover, the human enamel prism pattern that is established during the secretory stage of enamel development, was abnormal in a patient with a *RELT* mutation (Nikolopoulos et al., 2020). Previously our group demonstrated that ADAM10, but not ADAM17 can cleave the RELT extracellular domain (Ikeda et al., 2019). Therefore, ADAM10 may be an important modulator of RELT function that is necessary for proper enamel formation.

### Nectin1 and Nectin4

Nectins are a Ca^2+^-independent sub-family of immunoglobulin-like cell-cell adhesion molecules. All four nectins (NECTINS 1, 2, 3, and 4) are expressed within the mouse enamel organ by postnatal day 0 (Yoshida et al., 2010). Both NECTINS 1 and 4 are ADAM10 substrates (Kim et al., 2010; Buchanan et al., 2017).

Although *NECTIN1* mutations are not yet documented to cause enamel malformation in humans, deletion of *Nectin1* in mice causes their incisors to become hypomineralized. The maturation stage of development was shortened in these mice because during this stage the ameloblasts separated from the stratum intermedium and formed blister-like structures (Barron et al., 2008). Since ADAM10 is not expressed during the maturation stage of enamel development, it is unlikely that ADAM10 modulates NECTIN1 function during this stage.

Biallelic mutations in the human *NECTIN4* gene cause ectodermal dysplasia-syndactyly syndrome 1 (EDSS1). Two reports of EDSS1 had patients with associated enamel hypoplasia (Raza et al., 2015; Rotunno et al., 2021). Perhaps ADAM10 modulates NECTIN4 expression at the cell surface. However, ADAM17 also sheds NECTIN4 (Buchanan et al., 2017). Although it is unknown if ADAM17 is expressed on ameloblasts, it is expressed during the secretory stage in mouse molar enamel organs (Figure 2). Therefore, it remains possible that ADAM17 can substitute for the loss of ADAM10 in regulating NECTIN4 expression.

## Conclusion

ADAM10 has the possibility of performing several functions during enamel development. These possibilities include: ADAM10 may maintain the stem cell niche through Notch signaling so that the stem cells can properly develop into mature enamel organ cells. In support of this, a recent study showed that ADAM10-mediated Notch signaling is necessary for proper enamel formation (Mitsiadis et al., 2022). Once the stem cells become pre-ameloblasts, it is not known how the pre-ameloblasts begin to penetrate the basement membrane that separates them form the pre-dentin matrix. Although ADAM10 is expressed prior to and after basement membrane removal, the possibility exists that it could participate in its removal when paired with a tetraspanin that may promote its ability to cleave basement membrane proteins. Once murine incisor pre-ameloblasts become secretory stage ameloblasts, two types of ADAM10 function could promote the completion of the secretory stage of enamel development. First, ADAM10 could be directed to specific cell sites by tetraspanins such that it could separate the cohorts of ameloblasts that slide by one another to create the characteristic decussating enamel prism pattern (Figure 1). Therefore, this location-specific shedding of cell-cell contacts would separate the cells into cohorts, but would not separate the cells within a cohort. Second, ADAM10 can cleave the cell surface proteins COL17A1 and RELT that are present on ameloblasts during the secretory stage of enamel development. Both of these proteins are essential for proper enamel formation as their mutation was shown to cause enamel defects. Therefore, ADAM10 may be an important regulator of their function. Much remains to be characterized about the role of ADAM10 during enamel development to determine which, if any of these potential mechanisms of ADAM10 function are valid.
